# Functionalized Macrophage Exosomes with Panobinostat and PPM1D‐siRNA for Diffuse Intrinsic Pontine Gliomas Therapy

**DOI:** 10.1002/advs.202200353

**Published:** 2022-05-18

**Authors:** Shaobo Shan, Junge Chen, Yu Sun, Yongchao Wang, Bozhang Xia, Hong Tan, Changcun Pan, Guocan Gu, Jie Zhong, Guangchao Qing, Yuxuan Zhang, Jinjin Wang, Yufei Wang, Yi Wang, Pengcheng Zuo, Cheng Xu, Fangzhou Li, Weisheng Guo, Lijun Xu, Meiwan Chen, Yubo Fan, Liwei Zhang, Xing‐Jie Liang

**Affiliations:** ^1^ Key Laboratory for Biomechanics and Mechanobiology of Ministry of Education Beijing Advanced Innovation Center for Biomedical Engineering School of Biological Science and Medical Engineering & School of Engineering Medicine & Shenzhen Institute of Beihang University Beihang University Beijing 100083 P. R. China; ^2^ Department of Neurosurgery Beijing Tiantan Hospital Capital Medical University Beijing 100050 P. R. China; ^3^ CAS Key Laboratory for Biomedical Effects of Nanomaterials and Nanosafety CAS Center for Excellence in Nanoscience National Center for Nanoscience and Technology of China Beijing 100190 P. R. China; ^4^ Pediatric Epilepsy Center Peking University First Hospital No.1 Xi'an Men Street, Xicheng District Beijing 100034 P. R. China; ^5^ Department of Minimally Invasive Interventional Radiology College of Biomedical Engineering & The Second Affiliated Hospital Guangzhou Medical University Guangzhou 510260 P. R. China; ^6^ State Key Laboratory of Quality Research in Chinese Medicine Institute of Chinese Medical Sciences University of Macau Macau 999078 P. R. China; ^7^ China National Clinical Research Center for Neurological Diseases (NCRC‐ND) Beijing 100070 P. R. China

**Keywords:** blood–brain barrier, diffuse intrinsic pontine glioma, macrophage exosomes, panobinostat, siRNA blood‐brain barrier

## Abstract

Diffuse intrinsic pontine glioma (DIPG) is a rare and fatal pediatric brain tumor. Mutation of p53‐induced protein phosphatase 1 (PPM1D) in DIPG cells promotes tumor cell proliferation, and inhibition of PPM1D expression in DIPG cells with PPM1D mutation effectively reduces the proliferation activity of tumor cells. Panobinostat effectively kills DIPG tumor cells, but its systemic toxicity and low blood–brain barrier (BBB) permeability limits its application. In this paper, a nano drug delivery system based on functionalized macrophage exosomes with panobinostat and PPM1D‐siRNA for targeted therapy of DIPG with PPM1D mutation is prepared. The nano drug delivery system has higher drug delivery efficiency and better therapeutic effect than free drugs. In vivo and in vitro experimental results show that the nano drug delivery system can deliver panobinostat and siRNA across the BBB and achieve a targeted killing effect of DIPG tumor cells, resulting in the prolonged survival of orthotopic DIPG mice. This study provides new ideas for the delivery of small molecule drugs and gene drugs for DIPG therapy.

## Graphical abstract

Functionalized macrophage exosomes with panobinostat and PPM1D‐siRNA are constructed for targeted therapy of diffuse intrinsic pontine glioma (DIPG) with PPM1D mutation. The nano drug delivery system can deliver panobinostat and siRNA across the blood–brain barrier and achieve a targeted killing effect of DIPG tumor cells, resulting in the prolonged survival of orthotopic DIPG mice.

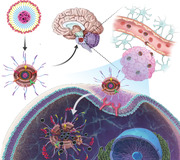

## 1 Introduction

Diffuse intrinsic pontine glioma (DIPG) is a rare and fatal brain tumor, accounting for 80% of pediatric brainstem gliomas (BSG). The median survival time is 9–12 months.^[^ ^1^ ^]^ The tumors exhibit strong invasiveness in the ventral side of pontine diffuse growth with the normal brainstem tissue, leading to limited treatment options. Because of the complex structure and critical functions of the brainstem, radical surgical resection can't be performed.^[^ ^2^ ^]^ Although radiotherapy temporarily relieves neurological symptoms, it cannot prolong the effective survival period of patients, and the prognosis of DIPG children is extremely poor.^[^ ^3^ ^]^ Therefore, drug therapy has become an important area of investigation.^[^ ^4^ ^]^

Existing studies have identified that nearly 90% of DIPG patients carry the histone H3 mutation H3K27M, and mutations in protein phosphatase, magnesium‐dependent 1, delta (PPM1D), tumor protein p53 (TP53), phosphatidylinositol‐4,5‐bisphosphate 3‐kinase, catalytic subunit alpha (PIK3CA), and activin A receptor type 1 (ACVR1) are also common.^[^ ^5^, ^6^, ^7^ ^]^ These somatic hypermutations are closely related with the occurrence and development of DIPG, and therefore they are expected to become therapeutic targets for DIPG.^[^ ^7^ ^]^ Our laboratory discovered the PPM1D mutation in DIPG for the first time. Subsequent studies have confirmed PPM1D mutation frequencies of up to 9%–25% in DIPG.^[^ ^6^, ^8^, ^9^, ^10^ ^]^ PPM1D mutation is also related to the chemotherapy resistance of myeloid cancer.^[^ ^11^ ^]^ We found that during clinical treatment, patients with brainstem glioma with PPM1D mutation have a shorter median survival time than PPM1D wild type patients. Studies have shown that the PPM1D‐mutated DIPG cells exhibit higher proliferation ability in both in vivo and in vitro experiments.^[^ ^12^, ^13^ ^]^ This is related to the increase structural stability and phosphatase activity of truncated PPM1D protein which promoting the dephosphorylation of PPM1D downstream substrates, such as p53 and *γ*‐H2AX, leading to inactivation of DNA damage response.^[^ ^14^ ^]^ Inhibition of mutated PPM1D can reduce tumor proliferation and increase apoptosis in PPM1D‐mutated DIPG cells.^[^ ^12^, ^14^ ^]^ Small interfering RNA (siRNA) has outstanding advantages and huge application potential in targeted cancer therapy.^[^ ^15^, ^16^, ^17^ ^]^ Several studies on brain tumors have shown that siRNA can specifically target pathogenic genes to make treatment more precise and personalized.^[^ ^18^, ^19^ ^]^ In this study, we designed siRNA against the mutated region of PPM1D. This siRNA specifically knocks down the expression of PPM1D in DIPG cells, thereby inhibiting tumor proliferation, which is in line with previous research reports.^[^ ^12^ ^]^

In addition, the histone deacetylase inhibitor‐panobinostat, which has been approved for the treatment of multiple myeloma by the FDA, can effectively inhibit the proliferation of DIPG cells.^[^ ^20^, ^21^ ^]^ The histone H3 mutation H3K27M is a characteristic gene mutation which exists in most DIPG patients. Studies have shown that panobinostat, one the most effective drugs for inhibiting the proliferation of DIPG cell lines, is effective not only against DIPG cells with histone H3 mutations but also against nonmutated cells. However, panobinostat has poor water solubility and low blood–brain barrier (BBB) penetration efficiency.^[^ ^22^ ^]^ Therefore, the administered dosage of panobinostat required to achieve an effective concentration in the central nervous system may cause intolerable systemic toxicity. This means that the application of panobinostat in DIPG is limited to a certain extent. During our research, we observed that siRNA‐mediated suppression of PPM1D expression in DIPG cells with PPM1D mutations not only inhibited cell growth in vitro but also increased the sensitivity of cells to panobinostat. Therefore, co‐delivery of panobinostat and PPM1D siRNA is expected to be an effective strategy for DIPG therapy. However, as mentioned above, panobinostat usually has high systemic toxicity, and siRNA is easily eliminated from the body. The specific delivery of toxic antagonists and fragile siRNA to tumor tissues remains a huge challenge.

Therapy with gene and small molecule drugs has good prospects for application in the clinical treatment of DIPG. However, due to the action of the BBB, the specificity of drug targeting is poor and the systemic toxicity is high. In the brainstem, delivery of drugs is limited by the complex local anatomy and the tight BBB.^[^ ^23^ ^]^ At the same time, the infiltration of the DIPG tumor into the local brainstem increases the anisotropy of the area and reduces the amount of chemotherapeutic drugs that can spread through the tumor area.^[^ ^24^ ^]^ At present, various synthetic nanocarriers have been used for tumor therapy.^[^ ^25^, ^26^, ^27^, ^28^ ^]^

Exosomes are nanovesicles with sizes of 30–150 nm which are secreted by living cells.^[^ ^29^, ^30^, ^31^, ^32^ ^]^ Some kinds of exosomes can successfully penetrate the blood–cerebrospinal fluid barrier.^[^ ^33^, ^34^ ^]^ Exosomes have^[^ ^28^ ^]^ the advantages of evading the host immune system and a long half‐life, and therefore they have a potential for the treatment of central nervous system diseases.^[^ ^34^, ^35^, ^36^, ^37^, ^38^, ^39^, ^40^ ^]^ Researchers from Oxford University first introduced the concept of using targeted modified exosomes for drug delivery. They used exosomes functionalized with the nerve‐specific RVG polypeptide to target siRNA to neurons for the treatment of Alzheimer's disease.^[^ ^34^ ^]^ Several researchers have loaded curcumin into exosomes and treated autoimmune encephalitis by nasal injection. They found that exosomes can quickly reach the brain tissue through the olfactory bulb and are taken up by macrophages to exert anti‐inflammatory effects.^[^ ^41^ ^]^ In addition, brain endothelial cell exosomes can carry antitumor drugs, which also proves that exosomes can smoothly pass through the blood–cerebrospinal fluid barrier to exert antitumor effects. Macrophage exosomes have been reported to successfully cross the blood–brain barrier.^[^ ^42^, ^43^, ^44^ ^]^ Macrophage exosomes carrying the integrin lymphocyte function‐associated antigen 1 (LFA‐1) can interact with intercellular adhesion molecule 1 (ICAM‐1) on cerebral vascular endothelial cells to mediate the lateral migration and exudation of macrophage exosomes, thereby crossing the blood–brain barrier.^[^ ^42^ ^]^ Engineering macrophage exosome encapsulating silica nanoparticles possesses efficient BBB penetration and good targeting capability for glioblastoma.^[^ ^45^ ^]^ Thus, macrophages exosomes have great application potential in the treatment of brainstem gliomas.

**Scheme 1:**
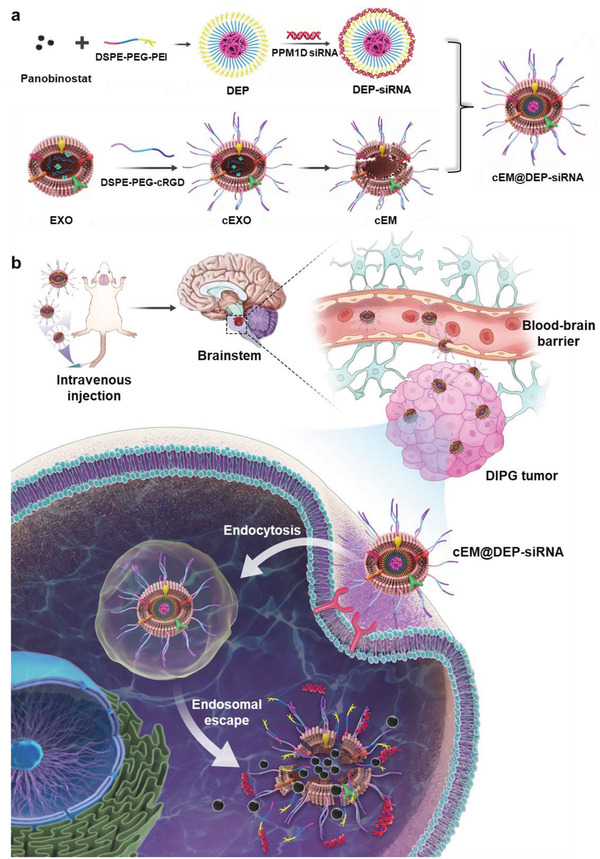
The design of exosome drug delivery system. a) Schematic illustration of the preparation of exosome drug delivery system (cEM@DEP‐siRNA). b) cEM@DEP‐siRNA was injected into DIPG xenograft animal models through tail vein. The nano drug penetrates the blood–brain barrier and specifically targeted the DIPG cells, which highly expressed integrin AV on the surface of tumor cells, then taken up by DIPG cells.

Based on the above research foundation, we have designed a biomimetic nano drug delivery system (cEM@DEP‐siRNA) which can efficiently co‐deliver panobinostat and PPM1D siRNA for treatment of DIPG with PPM1D mutation (**Scheme** **1**). Panobinostat and siRNA are complexed into positively charged micelles with high drug loading efficiency. The micelles are then surrounded by surface‐modified exosomal membranes to facilitate penetration of the BBB and targeting of DIPG tumors. Our in vitro and in vivo experiments confirm that the cEM@DEP‐siRNA nanoplatform can efficiently deliver gene and chemotherapy drugs, and inhibit the proliferation of DIPG cells.

## 2 Results and Discussion

### 2.1 Clinical Characteristics of DIPG

**Figure 1:**
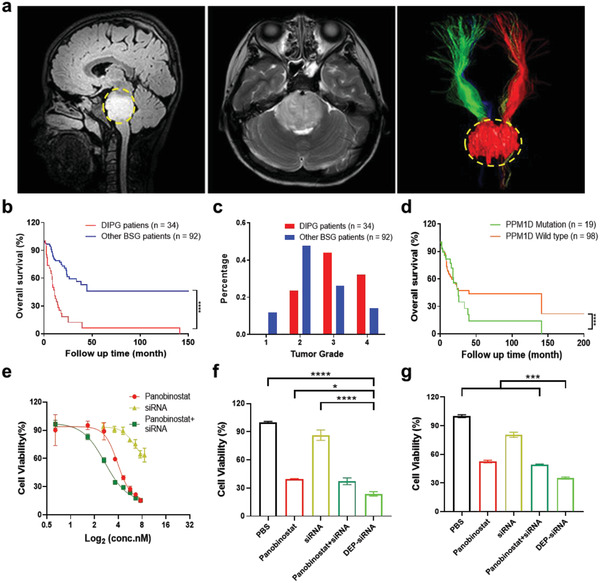
Clinical characteristics of DIPG with PPM1D mutation. a) Magnetic resonance imaging (MRI) 3D FLAIR sagittal (left) and T2 weighted axial (center) images of DIPG. Diffusion tensor imaging (DTI) 3D reconstruction results (right) of the corticospinal tract (CST) and the tumor displaying the infiltrative DIPG cells in the pons. The yellow dashed circle area indicates the normal pontine tissue invaded by the tumor, and the green and red fibrous images above the tumor area are the CST. b) Overall survival analysis of DIPG (*n* = 34) and non‐DIPG (*n* = 92) among 126 patients with brainstem glioma (BSG). c) Clinical information showing the percentage of DIPG and non‐DIPG histopathological grades. d) Survival analysis of 117 BSG patients with the PPM1D‐mutation (*n* = 19) or wild‐type PPM1D (*n* = 98). e) Viability of TT150714 DIPG cells after treatment with Panobinostat, siRNA, and Panobinostat+ siRNA at different concentrations for 72 h. f,g) Viability of TT150630 and TT150714 cells, respectively, after treatment with panobinostat, siRNA, panobinostat + siRNA, and DEP‐siRNA at a single concentration for 48 h. Panobinostat was administered at a concentration of 40 × 10^−9^ m; PPM1D siRNA was transfected at a dose of 100 × 10^−9^ m. The data show the mean ± s.e.m. from a representative experiment and analyzed by one‐way ANOVA, * *p* < 0.05, ** *p* < 0.01, *** *p* < 0.001, **** *p* < 0.0001.

The clinical characteristics of DIPG have been shown in **Figure** **1**. Figure 1a shows the MRI results from a typical case of DIPG in a child patient. The images show that the normal brainstem tissue is diffusely infiltrated by the tumor tissue, and the tumor has no clear boundary (Figure 1a and Figure S1a, Supporting Information). At the same time, the tumor tissue squeezes and destroys the normal nerve tract, which causes serious clinical symptoms. We evaluated the overall survival of 126 brainstem glioma (BSG) patients and found that DIPG patients exhibited shorter overall survival (11 months) compared to other BSG cases (Figure 1b). We collected tumor pathology information of the 126 patients with brainstem glioma. DIPG patients had a higher degree of tumor malignancy, with a higher proportion of grade III (15 out of 34, 44.1%) compared with other BSG patients (24 out of 92, 26.1%), and grade IV (11 out of 34, 32.4%) compared with other BSG patients (13 out of 92, 14.1%). Other BSG patients were mainly classified as grade II and III cases (II: 47.8%, III: 26.1%) (Figure 1c and Figure S1b, Supporting Information). This information shows that DIPG is more aggressive and malignant than other BSG, and DIPG patients have a shorter overall survival period compared with other BSG patients. When we compared the overall survival of BSG patients with and without PPM1D mutations, we found that patients with PPM1D mutations have a shorter overall survival time (Figure 1d).

Several studies have shown that PPM1D mutations in brainstem glioma can increase tumor proliferation and promote tumor cell growth. In our study, we explored the effects of different panobinostat and PPM1D siRNA formulations on the viability of two DIPG cells lines (TT150714 and TT150630) with PPM1D mutation. Three PPM1D siRNAs were designed to knock down PPM1D; the sequences of PPM1D siRNAs were listed in Table S1 (Supporting Information). PPM1D siRNA 2 was proven to have the best knockdown efficiency (74.35% ± 0.37%) compared with PPM1D siRNA 1 and 3 (Figure S2, Supporting Information). Therefore, PPM1D siRNA 2 was chosen for the following experiments. Then, we treated TT150714 with siRNA alone, panobinostat alone and panobinostat + siRNA. siRNA knockdown of PPM1D inhibited cell proliferation to a certain extent, and panobinostat alone had a strong inhibitory effect (Figure 1e). However, the inhibitory effect was significantly increased by the panobinostat + siRNA combination. This suggests that knockdown of PPM1D sensitized the TT150714 tumor cells to panobinostat.

Next, we constructed positively charged nanomicelles to simultaneously deliver panobinostat and PPM1D siRNA to obtain panobinostat‐loaded (DEP)‐siRNA nanomicelles. DEP was synthesized by a thin‐film hydration method, sealing panobinostat into the core of the micelles. The ratio of DSPE‐PEG‐PEI and panobinostat was varied to optimize the encapsulation efficiency of panobinostat. The optimal ratio was panobinostat:DSPE‐PEG‐PEI = 1:10 (Figure S3a,b, Supporting Information), and this ratio was used to prepare DEP. PPM1D siRNA was coated onto the surface of DEP through electrostatic adsorption (Figure S3c, Supporting Information) to obtain DEP‐siRNA. Among these treatments, the nanomedicine was most effective at inhibiting tumor cell proliferation (Figure 1f,g). The possible reason for this is that the nanocarrier increases the endocytosis efficiency of DIPG cells and improves the co‐delivery efficiency of small molecule drugs and gene drugs.

### 2.2 Preparation and Characterization of cEM@DEP‐siRNA

**Figure 2:**
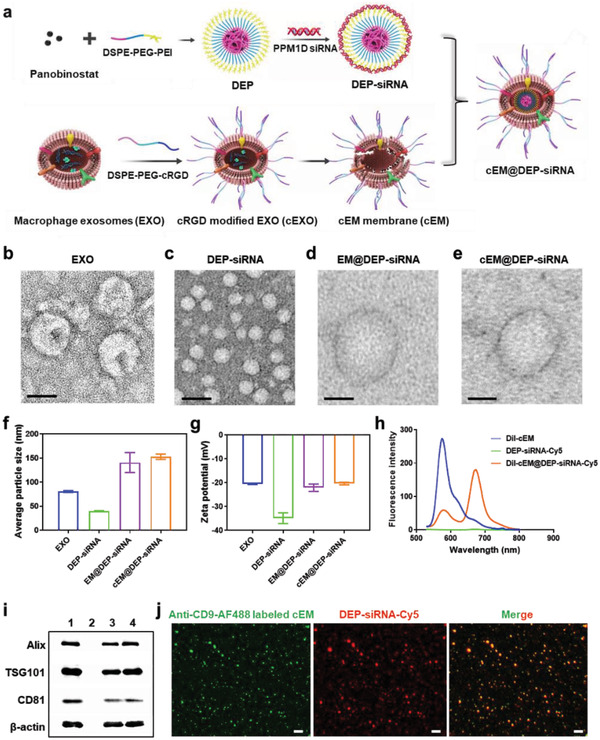
Characterization of the nano drug delivery system. a) Schematic illustration of the preparation of cEM@DEP‐siRNA. TEM images of b) EXO, c) DEP‐siRNA, d) EM@DEP‐siRNA, and e) cEM@DEP‐siRNA; Scale bar: 50 nm. f) Average particle size of EXO, DEP‐siRNA, EM@DEP‐siRNA, and cEM@DEP‐siRNA. g) Zeta potentials of EXO, DEP‐siRNA, EM@DEP‐siRNA, and cEM@DEP‐siRNA. h) Fluorescence intensity of DiI‐cEM, DEP‐siRNA‐Cy5, and DiI‐cEM@DEP‐siRNA‐Cy5. i) Western blot results of 1: EXO, 2: DEP‐siRNA, 3: EM@DEP‐siRNA, and 4: cEM@DEP‐siRNA. j) Co‐localization of cEM (labeled with anti‐CD9‐AF488, green) and DEP‐siRNA‐Cy5 (red) during the formation of cEM@DEP‐siRNA observed by Exoview R100; Scale bar: 2 µm.

In this study, we constructed a nano drug delivery system for tumor‐targeted co‐delivery of the chemotherapeutic drug panobinostat and the gene drug PPM1D siRNA (**Scheme** **1** and **Figure** **2**a). First, DEP were synthesized by thin‐film hydration method and coated with PPM1D siRNA through electrostatic adsorption. Next, macrophage exosomes (EXO) were isolated from RAW264.7 culture medium by ultracentrifugation. EXO carry LFA‐1 on the surface, which can interact with ICAM‐1 on cerebral vascular endothelial cells.^[^ ^42^ ^]^ This interaction mediates the lateral migration and exudation of macrophage exosomes, allowing them to cross the blood–brain barrier.^[^ ^42^ ^]^ EXO was modified with the tumor targeting peptide‐ cRGD, which could bind with integrin *α*_v_ *β*_3_ receptor on the surface of DIPG cells. Finally, the exosomal membranes were separately extracted from EXO and cEXO to get EM and cEM, and used to encapsulate DEP‐siRNA by a coextrusion process to achieve EM@DEP‐siRNA and cEM@DEP‐siRNA (Figure 2a).

Then, EXO, DEP‐siRNA, EM@DEP‐siRNA, and cEM@DEP‐siRNA were analyzed by transmission electron microscopy (TEM). EXO appeared as well‐defined membrane‐bound vesicles in TEM images (Figure 2b). DEP‐siRNA appeared as uniform micelles with clear boundaries (Figure 2c). EM@DEP‐siRNA and cEM@DEP‐siRNA both exhibited a typical phospholipid bilayer structure (Figure 2d,e). Figure 2f revealed that the mean size of cEM@DEP‐siRNA (152 ± 10 nm) was larger than that of EXO (80 ± 2 nm) and DEP‐siRNA (40 ± 2 nm) (Figure 2f and Figure S4, Supporting Information). Figure 2g showed that the zeta potentials of EM@DEP‐siRNA and cEM@DEP‐siRNA were similar with that of EXO. This proves that EM and cEM were successfully wrapped around the DEP‐siRNA micelles. The integrity of the cEM@DEP‐siRNA nanosystem was confirmed by fluorescence resonance energy transfer (FRET) measurements. The nanosystem was assembled using DEP‐siRNA carrying Cy5‐labeled PPM1D siRNA (DEP‐siRNA‐Cy5) and cEM labeled with 1,1″‐dioctadecyl‐3,3,3″,3′‐tetramethylindocarbocyanine perchlorate (DiI) (DiI‐cEM). Upon assembly of the nanosystem, the emission intensity of DiI‐cEM decreased at 590 nm and that of DEP‐siRNA‐Cy5 increased at 670 nm owing to FRET in intact DiI‐cEM@DEP‐siRNA‐Cy5 (Figure 2h). Furthermore, immunoblot analysis showed similar protein bands in EXO, EM@DEP‐siRNA and cEM@DEP‐siRNA (Figure 2i). Figure 2j reveals a notable colocalization between cEM, detected with AF488‐labeled anti‐CD9, and DEP‐siRNA‐Cy5 after the coextrusion process. This further demonstrates the integrity of cEM@DEP‐siRNA. The encapsulation efficiency and drug release profile of cEM@DEP‐siRNA is displayed in Figures S5 and S6 (Supporting Information). The stability of siRNA and cEM@DEP‐siRNA in 10% fetal bovine serum (diluted in PBS) for different time was detected. CEM@DEP‐siRNA was stable after incubated with 10% fetal bovine serum for 48 h (Figure S5b, Supporting Information). The panobinostat release of cEM@DEP‐siRNA after 24 h did not exceed 30% at pH 7.4 (Figure S6, Supporting Information), so cEM@DEP‐siRNA remained relatively stable at pH 7.4.

### 2.3 In Vitro BBB Transcytosis and Tumor Targeting Effect of Exosome Drug Delivery System

**Figure 3:**
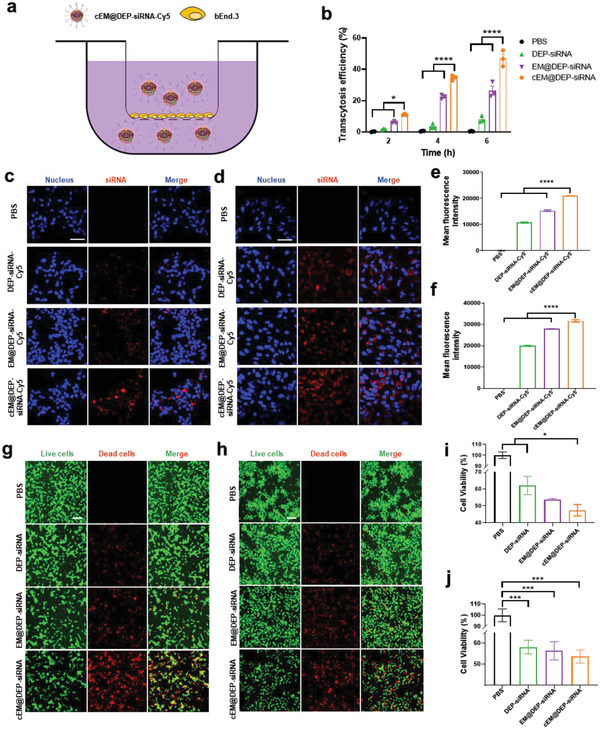
In vitro BBB transcytosis, cell uptake, and tumor killing effect of exosome drug delivery system. a) Illustration of the in vitro BBB model. b) Transcytosis efficiency of DEP‐siRNA, EM@DEP‐siRNA, and cEM@DEP‐siRNA in the in vitro BBB model. CLSM images and fluorescence intensity of c,e) TT150630 and d,f) TT150714 cells in different treatment groups, calculated by imageJ. The incubation time was 4 h. Scale bar: 50 µm. CLSM imaging of g) TT150630 and h) TT150714 DIPG cells after different treatments. Calcein‐AM (green fluorescence) labeled live cells and PI (red fluorescence) labeled dead cells. Scale bar: 200 µm. Cell viability in i) TT150630 and j) TT150714 cells in different treatment groups. Different treatment groups dosing the same drug concentration (panobinostat was administered at a concentration of 25 × 10^−9^ m). The incubation time was 72 h. The data show the mean ± s.e.m. from a representative experiment and analyzed by one‐way ANOVA, * *p* < 0.05, ** *p* < 0.01, *** *p* < 0.001, **** *p* < 0.0001.

To detect the BBB transcytosis ability of the exosome drug delivery system, an in vitro BBB model had been constructed of mouse brain‐derived endothelial cells (bEnd.3) (**Figure** **3**a). The BBB transcytosis ability of cEM@DEP‐siRNA‐Cy5 was significantly higher than that of DEP‐siRNA‐Cy5 and EM@DEP‐siRNA‐Cy5 (Figure 3b). Thus, cEM@DEP‐siRNA‐Cy5 could effectively traverse mouse BBB in vitro. To validate the tumor targeting capability of the biomimetic nano drug delivery system, the uptake of cEXO (labeled with DiI) by DIPG cells was evaluated by confocal laser scanning microscopy (CLSM). Figure S7 (Supporting Information) displayed that a large amount of cEXO was taken up into DIPG cells after co‐incubation for 4 h. In contrast, only a small amount of EXO was taken up by DIPG cells after co‐incubation for 4 h (Figure S7a, Supporting Information). Accordingly, the relative fluorescence intensity of DIPG cells incubated with DiI‐labeled cEXO was higher than that of the cells incubated with EXO (Figure S7b, Supporting Information). Thus, cEXO targets DIPG tumor cells more effectively than EXO. Next, the cellular uptake of DEP‐siRNA‐Cy5, EM@DEP‐siRNA‐Cy5, cEM@DEP‐siRNA‐Cy5 by DIPG cells was detected by CLSM. Figure 3c,d shows that more cEM@DEP‐siRNA‐Cy5 was taken up by TT150630 and TT150714 compared with DEP‐siRNA‐Cy5 and EM@DEP‐siRNA‐Cy5. Accordingly, the mean fluorescence intensity of TT150630 and TT150714 cells in the cEM@DEP‐siRNA‐Cy5 group was obviously higher compared with that in DEP‐siRNA‐Cy5 and EM@DEP‐siRNA‐Cy5 groups (Figure 3e,f). Thus, cEM@DEP‐siRNA‐Cy5 targets tumor cells more effectively than DEP‐siRNA‐Cy5 and EM@DEP‐siRNA‐Cy5. The ability of the nanosystem to escape from endosomes was tested by visualizing DEP‐siRNA‐Cy5 and cEM@DEP‐siRNA‐Cy5 in DIPG cells (TT150630, TT150714). Figure S8 (Supporting Information) shows that both DEP‐siRNA‐Cy5 and cEM@DEP‐siRNA‐Cy5 were separate from endosomes labeled with lysotracker green, proving that they could successfully achieve endosome escape.

### 2.4 In Vitro Cytotoxic Effect of Exosome Drug Delivery System

Next, the tumor killing effect of the biomimetic nano drug delivery system on TT150630 and TT150714 DIPG cells was explored. After incubation with different formulations for 72 h, Calcein AM/propidium iodide (PI) was used to detect live and dead cells and Cell‐titer@glo was used to quantitatively analyze cell viability. Among the different treatments, cEM@DEP‐siRNA had the strongest killing effect on DIPG cells (Figure 3g,h). Furthermore, the viability of TT150630 and TT150714 cells after 72 h incubation with PBS, DEP‐siRNA, EM@DEP‐siRNA, and cEM@DEP‐siRNA was evaluated. Treatment with cEM@DEP‐siRNA induced higher cytotoxicity than the other formulations (Figure 3i,j and Figure S9a,b, Supporting Information). In addition, DIPG cell apoptosis induced by different treatments was detected by flow cytometry. As displayed in Figure S16 (Supporting Information), the cEM@DEP‐siRNA treated group presented more severe early and late apoptosis than the other groups. These results suggest that cEM@DEP‐siRNA can enhance the inhibitory effect of panobinostat and PPM1D siRNA. The relative expression levels of PPM1D mRNA in TT150630 and TT150714 was evaluated by RT‐PCR. The relative PPM1D expression level was lowest after treatment by cEM@DEP‐siRNA (Figure S10, Supporting Information). Thus, cEM@DEP‐siRNA had the best knockdown efficiency.

### 2.5 Blood Pharmacokinetics and In Vivo Biodistribution of Exosome Drug Delivery System

**Figure 4:**
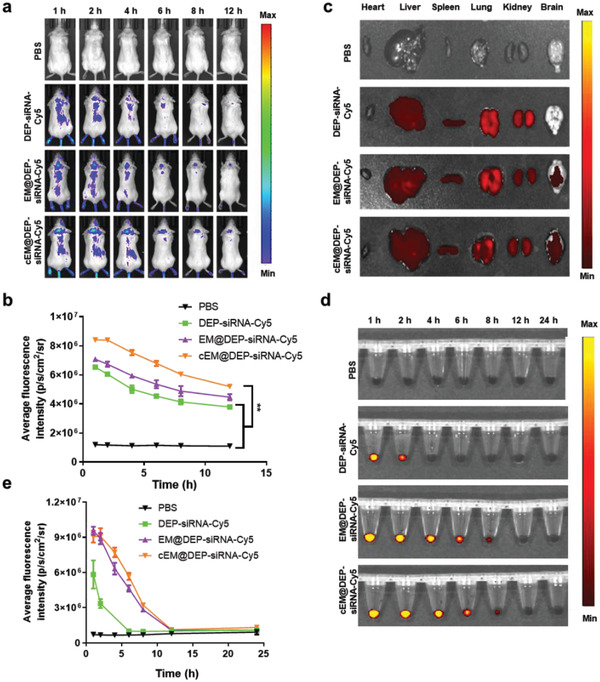
In vivo biodistribution and blood pharmacokinetics of exosome drug delivery system. a) In vivo distribution of PBS, DEP‐siRNA‐Cy5, EM@DEP‐siRNA‐Cy5, and cEM@DEP‐siRNA‐Cy5 in orthotopic DIPG‐bearing mice (*n* = 3). b) Average fluorescence intensities of mice's brain after treatment by PBS, DEP‐siRNA‐Cy5, EM@DEP‐siRNA‐Cy5, and cEM@DEP‐siRNA‐Cy5 for different time. c) Ex vivo fluorescent images of major organs at 12 h postinjection. d) Fluorescence images and e) average fluorescence intensities of blood samples collected from mice, after treatment by PBS, DEP‐siRNA‐Cy5, EM@DEP‐siRNA‐Cy5, and cEM@DEP‐siRNA‐Cy5, at the indicated time points (1, 2, 4, 6, 8, 12, and 24 h) post administration. The data show the mean ± s.e.m. from a representative experiment and analyzed by one‐way ANOVA, * *p* < 0.05, ** *p* < 0.01, *** *p* < 0.001, **** *p* < 0.0001.

Based on the good tumor cell‐targeting ability of the nano drug delivery system in vitro, orthotopic DIPG‐bearing mice were constructed (Figure S11, Supporting Information), with the aim of investigating the in vivo biodistribution of the nanosystem and its capacity to cross the BBB. After intravenous injection of PBS or nanoparticles (panobinostat = 0.2 mg kg^−1^; PPM1D‐siRNA‐Cy5 = 1 mg kg^−1^ for each mouse), fluorescence images were obtained at different time points with an IVIS Spectrum system. The fluorescence signals in the head of the cEM@DEP‐siRNA‐Cy5 group were higher than the fluorescence signals in the DEP‐siRNA‐Cy5 and EM@DEP‐siRNA‐Cy5 groups (**Figure** **4**a,b). 12 h postinjection, the main organs in each group were dissected for ex vivo fluorescence imaging. The results indicated that cEM@DEP‐siRNA‐Cy5 was distributed mainly in the liver, lung, kidney, and brain (Figure 4c). Fluorescence signals in the brain in the cEM@DEP‐siRNA‐Cy5 group were higher than the fluorescence signals in the DEP‐siRNA‐Cy5 and EM@DEP‐siRNA‐Cy5 groups (Figure 4c and Figure S12, Supporting Information). This indicates that cEM@DEP‐siRNA‐Cy5 has the highest capacity to cross the BBB. Then the blood pharmacokinetics of exosome drug delivery system was investigated. Blood samples were collected from healthy female BALB/c mice after intravenous injection of DEP‐siRNA‐Cy5, EM@DEP‐siRNA‐Cy5, cEM@DEP‐siRNA‐Cy5 for different times, and analyzed by IVIS Spectrum system for fluorescence imaging and quantification. Fluorescent signals were highest at the initial time and then gradually decreased. DEP‐siRNA‐Cy5 could not be detected at 4 h. However, EM@DEP‐siRNA‐Cy5 and cEM@DEP‐siRNA‐Cy5 could be detected in mouse blood until 8 h. We could see that EM@DEP‐siRNA‐Cy5 and cEM@DEP‐siRNA‐Cy5 displayed significantly prolonged circulation compared to DEP‐siRNA‐Cy5 (Figure 4d,e). Therefore, cEM@DEP‐siRNA has great potential in the treatment of brain tumors.

### 2.6 In Vivo Therapeutic Effect of cEM@DEP‐siRNA

**Figure 5:**
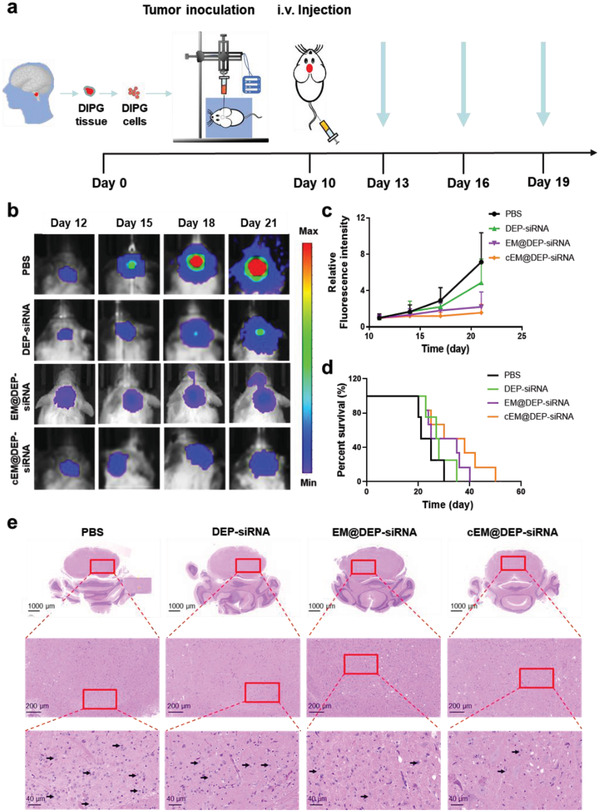
Antitumor effect of exosome drug delivery system in the orthotopic DIPG model. a) Schematic of the treatment process. b) IVIS biofluorescence imaging of representative DIPG tumor‐bearing mice in each treatment group. c) Bioluminescence signal intensity curves of the different treatment groups (*n* = 6). d) Kaplan–Meier survival curves of mice after treatment with PBS, DEP‐siRNA, EM@DEP‐siRNA, and cEM@DEP‐siRNA. e) H&E staining of the tumor tissues from pons of mice after treatment with PBS, DEP‐siRNA, EM@DEP‐siRNA, and cEM@DEP‐siRNA. Tumor cells were labeled with small black arrow.

Orthotopic DIPG animal models were constructed to investigate the antitumor efficacy of cEM@DEP‐siRNA. The process for constructing the animal model is shown in **Figure** **5**a and Figure S11 (Supporting Information). Fluorescence imaging was used to detect the signal in the tumor, and mice with a similar intensity of tumor fluorescence were randomly divided into four groups. We injected different treatments into the tail vein every 3 d for four consecutive injections. The bioluminescence images were taken every 3 d to monitor tumor growth. We found that the antitumor proliferation effect of cEM@DEP‐siRNA (the dosage of panobinostat was 0.5 mg kg^−1^, the dosage of PPM1D‐siRNA was 2.5 mg kg^−1^ i.v.) was superior to the antitumor proliferation effect of EM@DEP‐siRNA (panobinostat = 0.5 mg kg^−1^, PPM1D‐siRNA = 2.5 mg kg^−1^, i.v.), DEP‐siRNA (panobinostat = 0.5 mg kg^−1^, PPM1D‐siRNA = 2.5 mg kg^−1^, i.v.) and PBS (Figure 5b,c). This indicates that the physicochemical properties of cEM@DEP‐siRNA are key factors in enhancing the therapeutic efficacy in vivo.

The tumor suppression effect in the cEM@DEP‐siRNA group (panobinostat = 0.5 mg kg^−1^, PPM1D‐siRNA = 2.5 mg kg^−1^, i.v.) was similar to that in the group receiving a high‐dose intraperitoneal injection of free panobinostat (5.0 mg kg^−1^, i.p.) (Figure S13, Supporting Information). In the overall survival analysis, the cEM@DEP‐siRNA group presented the best overall survival out of all the treatment groups (Figure 5d), and had a similar overall survival time as the group receiving high‐dose free panobinostat (5.0 mg kg^−1^, i.p.) via intraperitoneal injection (Figure S14, Supporting Information). This shows that cEM@DEP‐siRNA (panobinostat = 0.5 mg kg^−1^, PPM1D‐siRNA = 2.5 mg kg^−1^, i.v.) can achieve the therapeutic effect of a high dose of free panobinostat (5.0 mg kg^−1^, i.p.). Mice treated with cEM@DEP‐siRNA showed fewer pleomorphic tumor cells by tumor hematoxylin and eosin (H&E) staining, compared to the other groups (Figure 5e). These results indicate that cEM@DEP‐siRNA effectively crosses the BBB and penetrates into DIPG tissues. Such properties are critical factors for successful DIPG treatment.

### 2.7 Biological Safety of Exosome Drug Delivery Systems

**Figure 6:**
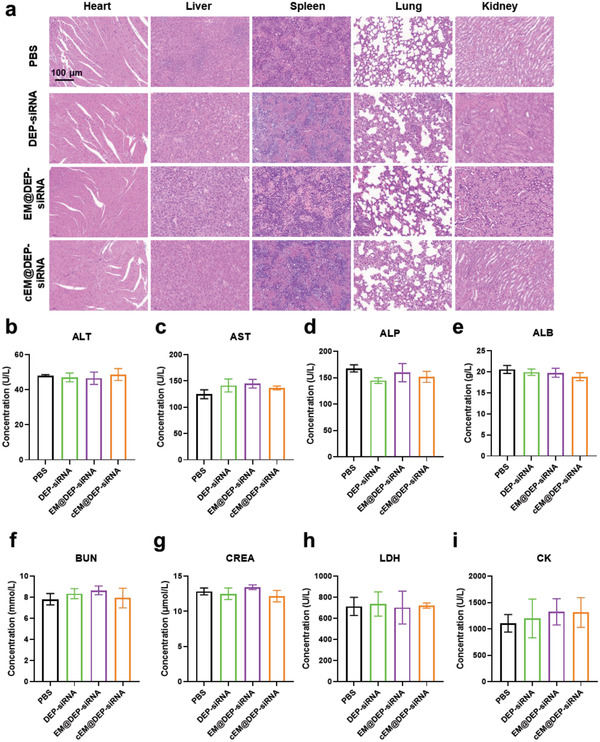
Biological safety of exosome drug delivery systems. a) Images of H&E‐stained heart, liver, spleen, lung, and kidney from mice in PBS, DEP‐siRNA, EM@DEP‐siRNA, and cEM@DEP‐siRNA groups. Scale bar: 100 µm. b–i) Toxicity of the nanomedicines evaluated by blood biochemical index level. Liver function indicators include (b) alanine transaminase (ALT), (c) aspartate transaminase (AST), (d) alkaline phosphatase (ALP), and (e) albumin (ALB). Kidney function indicators include (f) blood urea nitrogen (BUN) and (g) creatinine (CREA). Heart function indicators include (h) lactate dehydrogenase (LDH) and (i) creatine kinase (CK).

To further evaluate the application potential of our biomimetic nano drug delivery systems in biomedicine, we tested the biosafety of the formulation in vivo. We found that there was no obvious change in the tissue structure of the major organs (**Figure** **6**a), or in indexes of liver, kidney, and heart function (Figure 6b–i). Moreover, the body weight of mice in PBS, DEP‐siRNA, EM@DEP‐siRNA, and cEM@DEP‐siRNA groups showed no significant changes (Figure S15, Supporting Information). These results suggest that the nanocarrier treatments are safe for DIPG therapy.

## 3 Conclusion

In summary, this study has successfully developed a functionalized macrophage exosome‐based nano drug delivery system loaded with panobinostat and PPM1D siRNA for the targeted therapy of DIPG. In vitro, this cEM@DEP‐siRNA system effectively kills DIPG tumor cells. By in vivo experiment, cEM@DEP‐siRNA achieves significant tumor growth inhibition and prolonged survival time in orthotopic DIPG‐bearing mice. This can be ascribed to the high BBB penetration efficacy and good tumor targeting capability. The antitumor mechanism of cEM@DEP‐siRNA is as follow: after administered to DIPG orthotopic xenograft model by tail intravenous injection, cEM@DEP‐siRNA could penetrate the blood–brain barrier by the function of EXO, and accumulates at the tumor site and uptaken by DIPG cells by cRGD, then release panobinostat and PPM1D siRNA by endosome escape. Therefore, this paper provides theoretical and technical reference for the treatment of DIPG and other central nervous system tumors.

## 4 Experimental Section

### Patient Cohort Characteristics Collection

The patients’ information including MRI and clinical data in this study are from the Department of Neurosurgery, Beijing Tiantan Hospital (Beijing, China). The previous article of this team also reported other clinical characteristics and gene sequencing information of these patients.^[^ ^46^ ^]^ The corticospinal tract (CST) tracts were reconstructed using the fiber association by continuous tractography (FACT) method with Pipeline for Analyzing Brain Diffusion Images (Medtronic workstation). DIPG were graded according to the CNS WHO classification. Kaplan–Meier analysis and log‐rank test were used for survival analysis.

### Primary DIPG Cell Culture and Cell Viability

The patient‐derived DIPG tumor cell lines (TT150630, TT150714) were established before, and confirmed the PPM1D gene mutation, used in several DIPG studies.^[^ ^4^, ^13^ ^]^ Tumor cells were cultured with DIPG medium in a plate coated with 1% Matrigel. DIPG medium contains DMEM, N2 (100×), B27 (50×), EGF (20 ng mL^−1^), and bFGF (20 ng mL^−1^). DIPG cells were cultured in a Thermo Company Forma Series II CO_2_ constant temperature cell incubator with 37 °C and 5% CO_2_. CellTiter‐Glo was used to measure cell viability by the manufacturer's protocol. A multimode microplate detection system (Enspire) was used to measure luminescence signals.

### Purification of Exosomes

RAW264.7 cells were cultured in DMEM total medium until the cell confluence reached 70%–80%. Cells were washed by PBS for three times and cultured in plasma‐free DMEM medium for 48 h. The cell culture medium was collected for exosome purification. Then, macrophage exosomes were isolated according to the previous report.^[^ ^47^ ^]^ The details were as follows: First, the collected macrophage cell culture medium was centrifuged at 3000 *g* for 10 min to remove cells. After that, the supernatant was centrifuged at 10 000 *g* for 30 min. And the supernatant was concentrated by concentrators (100 kDa, 15 mL) and filtered with a 0.22 µm filter. Then the filtrate was centrifuged at 160 000 *g* for 70 min. And the exosome pellet was washed by PBS and centrifuged at 160 000 *g* for 70 min (4 °C) one more time. Finally, the exosomes were resuspended by PBS and stored at −20 °C until use.

### Panobinostat and siRNA Loading

The synthesis of cEM@DEP‐siRNA requires three steps. In step one, panobinostat was loaded into the core of positively charged nanomicelles generated from DSPE‐PEG_2000_‐PEI_25000_ via the filming‐rehydration method. In brief, panobinostat and DSPE‐PEG_2000_‐PEI_25000_ were dissolved in an appropriate amount of organic solvent (chloroform:anhydrous methanol = 1:1). Then the panobinostat solution and the DSPE‐PEG_2000_‐PEI_25000_ solution were mixed uniformly (the mass ratio of DSPE‐PEG_2000_‐PEI_25000_ to panobinostat was 10:1). The mixed solution was then transferred into an evaporation flask, and the organic solvent was removed by a vacuum rotary evaporator. A dry lipid film containing panobinostat was formed at the bottom of the bottle. Then an appropriate amount of PBS buffer was added to dissolve the lipid membrane, and the membrane was hydrated in a 60 °C water bath for 30 min. The hydrated solution was filtered by a 0.22 µm filter to remove free panobinostat. This yielded the DEP micellar solution. In step two, the siRNA was coated on the surface of DEP by electrovalent interaction. DEP micelles were incubated with PPM1D siRNA in deionized water for 1 h. To obtain a suitable mass ratio of DSPE‐PEG‐PEI to siRNA, the same amount of siRNA was incubated with solutions containing different concentrations of DEP. The mass ratios of DEP to siRNA were 100:512, 100:256, 100:128, 100:64, 100:32, 100:16, and 100:8. The agarose gel retardation assay was used to study the binding of DEP and siRNA and the optimum mass ratio of DEP to siRNA was selected for preparation of DEP‐siRNA.

### Preparation of cEM@DEP‐siRNA

First, RAW264.7 exosomes (EXO), prepared as described above, were modified with DSPE‐PEG‐cRGD to get cRGD‐functionalized EXO (cEXO) according to a previous report.^[^ ^35^ ^]^ The details were as follows: DSPE‐PEG_2000_‐cRGD was dissolved in HEPES buffer at 60 °C for 15 min. Then, EXO were mixed with DSPE‐PEG_2000_‐cRGD in a 1:1 ratio and incubated at 40 °C for 2 h. After that, the above mixtures were cooled to 4 °C. And free DSPE‐PEG_2000_‐cRGD was removed by ultrafiltration. Then the exosomal membrane was extracted from unmodified EXO and cEXO by incubation with low permeable solution buffer (containing protease inhibitor cocktail) overnight, followed by ultracentrifugation (160 000 *g*, 70 min) to get exosomal membranes from EXO (EM) and cEXO (EM). Finally, EM and cEM were used to encapsulate DEP‐siRNA by coextrusion via a liposome extruder (220 µm, 12 times) to get EM@DEP‐siRNA and cEM@DEP‐siRNA. To evaluate the ability of cEM@DEP‐siRNA to protect the siRNA from degradation, naked siRNA and cEM@DEP‐siRNA with a mass ratio of panobinostat/siRNA of 1:1 was added to 10% fetal bovine serum in PBS.

### Characterization of cEM@DEP‐siRNA

TEM was used to characterize the morphology of the nanoparticles. The details are as follows: 7 µL nanoparticles were dropped onto copper grids and incubated for 2 min. After that, they were removed by filter paper. Then, nanoparticles were negatively stained by 2% uranyl acetate for 2 min. Finally, the nanoparticles were observed by HT7700 (Hitachi, Japan). The size distribution of the nanoparticles was acquired by a Malvern Zetasizer (Nano ZSP 3600, England) after a 50‐fold dilution. Western blotting was used for protein analysis as follows: 20 µg of nanoparticles were loaded per well of a 4%–15% Tris‐glycine PAGE Precast‐Glgel (Sangon Biotech, China) and separated by electrophoresis at 160 V for 60 min. Then proteins were transferred onto a nitrocellulose (NC) membrane (Pall Corporation, USA) by transferring at 300 mA for 90 min. Then the NC membrane was blocked by 5% skim milk powder for 60 min. Primary antibodies including anti‐CD81 (Cell Signaling, USA), anti‐TSG101 (Abcam, England), anti‐Alix (Cell Signaling, USA), and anti‐*β*‐actin (Cell Signaling, USA) were incubated with the NC membrane at 4 °C overnight, and washed with TBST for three times, incubated with secondary antibodies (anti‐rabbit HRP or anti‐mouse HRP (Beyotime, China)) for 1 h and washed three times in TBST. Finally, target proteins were detected by incubating the membrane with enhanced chemiluminescence reagents (Thermo, USA).

### Reverse Transcription qPCR

The total RNA of DIPG cells was extracted using a RNeasy kit (Qiagen, German) according to the kit protocol. And cDNA was acquired using a First Strand cDNA Synthesis kit (Thermo, USA) according to the kit protocol. QPCR reaction systems containing 10 µL 2* qPCR SYBR master mix (Yeasen, China), 0.4 µL of each primer, 2 µL of each cDNA preparation, and 7.6 µL ddH_2_O. qPCR was performed at 95 °C for 2 min, then 95 °C for 10 s, and 60 °C for 40 s on a quantitative PCR instrument (Archimed X6, ROCGENE, China). The sequences of PPM1D siRNA are given in Table S1 (Supporting Information).

### Drug Encapsulation Efficiency and Drug Release

To detect the content of panobinostat in the nanoparticles, newly prepared nanoparticles were concentrated by concentrators (100 kDa) and the filtrate was analyzed by measuring the absorbance at 277 nm using a UV–vis spectrophotometer. The content of encapsulated panobinostat was calculated by subtracting the unencapsulated panobinostat from the initial mass of drug. The formulas for calculating encapsulation efficiency and loading efficiency of panobinostat are as follows

(1) $$\text{Encapsulation}\;\text{efficiency}\;\left(\%\right)=M_{E}/M_{I}\times 100$$

where *M*_E_ means the mass of panobinostat encapsulated in nanoparticles and *M*_I_ means the initial mass of panobinostat.

To determine the drug release profile, 1 mL DEP‐siRNA and cEM@DEP‐siRNA suspensions were, respectively, dialyzed in 10 mL PBS (pH 6.5) and PBS (pH 7.4) in a dialysis bag (10 kDa) at room temperature with orbital shaking at about 200 rpm. At 1, 2, 4, 9, 48, and 72 h, 1 mL dialysis buffer was collected for panobinostat quantification. At the same time, another 1 mL PBS was added into the system. Then, the released panobinostat was quantified by UV–vis spectrometry.

### Cell Uptake

To evaluate the cell uptake of nanoparticles, 1.0 × 10^4^ TT150630 or TT150714 cells were seeded into a confocal eight‐well dish with DIPG medium, and cultured at 37 °C overnight. After that, PBS, DEP‐siRNA‐Cy5, EM@DEP‐siRNA‐Cy5, or cEM@DEP‐siRNA‐Cy5 was incubated with the cells at 37 °C for 4 h. Then the cell medium was discarded, and DIPG cells were washed with 200 µL PBS for three times to remove all the nanoparticles. The cells were fixed with 4% paraformaldehyde at 4 °C for 10 min, stained by 200 µL DAPI solution for 10 min, then washed by 200 µL PBS to remove the free DAPI. Finally, the uptake of DEP‐siRNA‐Cy5, EM@DEP‐siRNA‐Cy5, or cEM@DEP‐siRNA‐Cy5 by TT150630 and TT150714 cells was observed using a LCSM (CLSM, Zeiss, Japan).

### Transcytosis Assay by In Vitro BBB Model

Mouse brain endothelial bEnd.3 cells were used to construct the in vitro BBB model. The bEnd.3 cells were seeded into 0.4 µm transwell plates (24 well Transwell, Corning Incorporated) at a density of 1 × 10^6^ cells/well, and the cells were cultured in fresh medium for 72 h. When the trans‐endothelial electrical resistance of the model was greater than 300 Ω indicates that the BBB model is successfully constructed. Add the Cy5 labeled nanocarrier to the top chamber. Then collect samples from the bottom chamber at the time point of 2, 4, and 6 h, respectively. And the multimode microplate detection system (Enspire) was used to detect the corresponding fluorescence values of each group samples.

### Intracellular Drug Release and Endosome Escape

To detect the intracellular drug release and endosome escape of nanoparticles, 1.0 × 10^4^ TT150630 or TT150714 cells were seeded into a confocal eight‐well dish with DIPG medium, and cultured at 37 °C overnight. After that, PBS, siRNA labeled by Cy5 (siRNA‐Cy5) and cEM@DEP‐siRNA‐Cy5 was separately incubated with the cells at 37 °C for 4 h. Then the cell medium was discarded and TT150630 or TT150714 cells were washed three times with 200 µL PBS to remove all the nanoparticles. Cells were stained with Lysotracker Green (to label endosomes) and Hoechst (to label nuclei) for 20 min. Finally, the intracellular drug release and endosomal escape was observed by a LCSM (with ZEN 2010 software).

### DIPG Orthotopic Animal Models and In Vivo Antitumor Efficacy

All animal experiments were conducted in accordance with the guidelines provided by the National Center for Nanoscience Animal Health and Use Committee (NCNST21‐2104‐0405). The orthotopic DIPG mouse model was established in female NCG(NOD/ShiLtJGpt‐Prkdc^em26^Il2rg^em26^/Gpt) mice (Jiangsu Jicuiyaokang, China). 100 000 DIPG cells with Luciferase gene were suspended in 5 µL PBS and implanted into the brainstem of the mice as previously described operation steps.^[^ ^48^ ^]^ After 10 d, fluorescence imaging of the mice was carried out to determine the success of the tumor inoculation. Then the mice successfully inoculated with tumors were numbered and divided into four groups according to the intensity of the fluorescence signal. The tumor‐bearing mice were treated as follows: PBS (control), DEP‐siRNA, EM@DEP‐siRNA, and cEM@DEP‐siRNA at 0.5 mg kg^−1^ panobinostat and 2.5 mg kg^−1^ siRNA via tail vein injection (*n* = 6). The procedure was repeated every 3 d. Each mouse received a total of four injections. During the therapy, the body weight of mice was recorded every 3 d. Bioluminescence imaging was performed on DIPG orthotopic model every 3 d using an IVIS Spectrum imaging system (PerkinElmer, USA). Each mouse was imaged four times. Bioluminescence signal intensity was measured by Living Image 4.4 software (PerkinElmer, USA).

### In Vivo Biodistribution and Blood Pharmacokinetics

For in vivo biodistribution and blood pharmacokinetics analysis, DEP‐siRNA‐Cy5, EM@DEP‐siRNA‐Cy5, and cEM@DEP‐siRNA‐Cy5 were injected into NCG mice via the tail vein. The dosage of panobinostat was 0.5 mg kg^−1^ in DEP‐siRNA‐Cy5, EM@DEP‐siRNA‐Cy5, and cEM@DEP‐siRNA‐Cy5. Then 10 µL blood sample was collected by tail snip after treatment for different time (1, 2, 4, 6, 8, 10, 12 and 24 h), and analyzed by ISIV system to detect Cy5 signal (650 excitation/670 nm emission) for blood pharmacokinetics analysis. To evaluate the in vivo biodistribution of nanoparticles, PBS, DEP‐siRNA‐Cy5, EM@DEP‐siRNA‐Cy5, and cEM@ DEP‐siRNA‐Cy5 were injected into NCG mice via the tail vein. Then, fluorescent images were captured at 1, 5, 7, 12, and 24 h using IVIS Spectrum (PerkinElmer, USA).

### Biosafety Evaluation

On the 15th day, blood was drawn for hematologic analysis. The major organs were fixed in 10% formalin, embedded in paraffin, and sliced into 8 µm sections for H&E staining.

## Conflict of Interest

The authors declare no conflict of interest.

## Author Contributions

S.S. and J.C. contributed equally to this work. S.S., J.C., L.X., and L.Z. designed and completed the experiments. S.S., J.C., Y.S., C.P., G.G., Y.W., P.Z., C.X., Y.W., B.X., H.T., J.Z., G.Q., Y.Z., J.W., Y.W., F.L., W.G., M.C., Y.F., and X.‐J.L. collected and analyzed the data. S.S. and J.C. wrote the manuscript.

## Supporting information

Supporting Information

## Data Availability

The data that support the findings of this study are available in the supplementary material of this article.
