# Supplementary material for: Functionalized Macrophage Exosomes with Panobinostat and PPM1D‐siRNA for Diffuse Intrinsic Pontine Gliomas Therapy
Source: Adv Sci (Weinh). 2022 May 18;9(21):2200353. doi: 10.1002/advs.202200353 (PMC9313473; doi:10.1002/advs.202200353)
Supplement: Supplementary file 1 — Supporting Information [file ADVS-9-2200353-s001.pdf]

Supporting Information

Functionalized Macrophage Exosomes with Panobinostat and PPM1D-siRNA for Diffuse Intrinsic Pontine Gliomas Therapy

Shaobo Shan[1,2,3,#], Junge Chen[1, 3,#], Yu Sun[4], Yongchao Wang[2,3], Bozhang Xia[3], Hong Tan[3], Changcun Pan[2], Guocan Gu[2], Jie Zhong[3], Guangchao Qing[3], Yuxuan Zhang[3], Jinjin Wang[3], Yufei Wang[3], Yi Wang[2], Pengcheng Zuo[2], Cheng Xu[2], Fangzhou Li[3], Weisheng Guo[7], Lijun Xu[1], Meiwan Chen[5], Yubo Fan[1], Liwei Zhang[1,2,6,#], Xing-Jie Liang[3,]*

[1] Key Laboratory for Biomechanics and Mechanobiology of Ministry of Education| Beijing Advanced Innovation Center for Biomedical Engineering, School of Biological Science and Medical Engineering & School of Engineering Medicine & Shenzhen Institute of Beihang University, Beihang University, Beijing 100083, P. R. China

[2] Department of Neurosurgery, Beijing Tiantan Hospital, Capital Medical University, Beijing100050, P. R. China

[3] CAS Key Laboratory for Biomedical Effects of Nanomaterials and Nanosafety, CAS Center for Excellence in Nanoscience, National Center for Nanoscience and Technology of China, Beijing, 100190, P. R. China.

[4] Pediatric Epilepsy Center, Peking University First Hospital, No.1 Xi'an Men Street, Xicheng District, Beijing 100034, P. R. China

[5] State Key Laboratory of Quality Research in Chinese Medicine, Institute of Chinese Medical Sciences, University of Macau, Macau 999078, P. R. China

[6] China National Clinical Research Center for Neurological Diseases (NCRC-ND), Beijing 100070, P. R. China

[7] Department of Minimally Invasive Interventional Radiology, College of Biomedical Engineering & The Second Affiliated Hospital, Guangzhou Medical University, Guangzhou 510260, P. R. China

[#] These authors contributed equally to this work.

* Corresponding author.

E-mail: zhangliweittyy@163.com; liangxj@nanoctr.cn

a

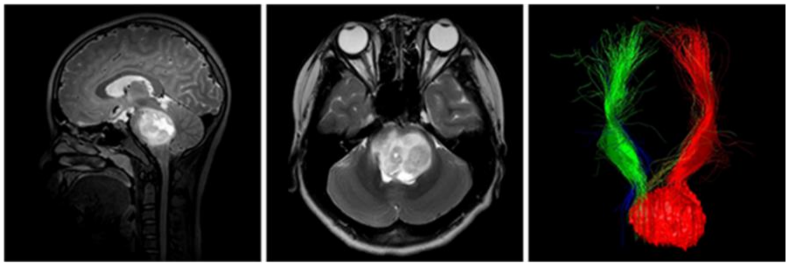

b

BSG Grade	I	II	III	IV	Total
DIPG	0	8	15	11	34
Other BSG	11	44	24	13	92

Figure S1. Clinical information of DIPG patients. (a) MRI T2 weighted sagittal, axial images, and the DTI three-dimensional reconstruction results of CST and tumor in different patients. (b) The ratio of DIPG and other BSG histopathological grades.

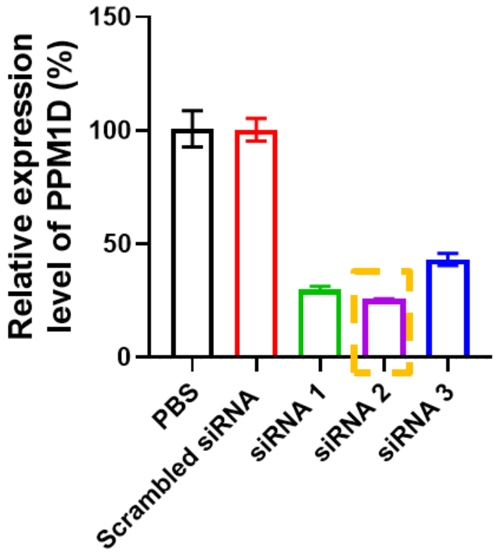

Figure S2. **Knockdown efficiency of PPM1D siRNA 1, siRNA 2, and siRNA 3.** siRNA 2 displayed the best knockdown efficiency.

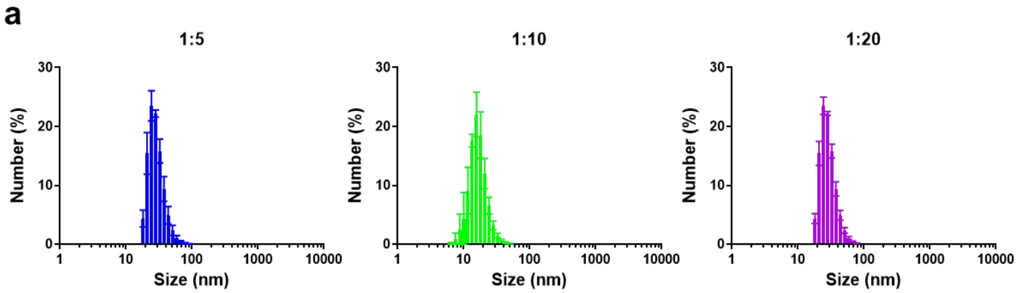

b

Panobinostat: DSPE-PEG-PEI	Diameter (nm)	PDI	Encapsulation efficiency	ζ-potential (mV)
**1:5**	34.16±2.57	0.49	40.76%	31.60±1.25
**1:10**	18.24±0.90	0.55	78.71%	33.23±1.56
**1:20**	17.02±2.88	0.54	58.77%	20.90±0.95

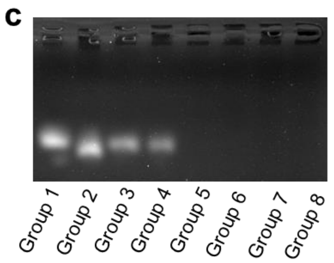

Figure S3. Optimization of micelle synthesis conditions. (a) Size distribution of DEP-siRNA with Pano:DPP= 1:5, 1:10, and 1:20 (m/m). (b) Encapsulation efficiency and ζ-potential of DEP-siRNA with Pano:DPP = 1:5, 1:10, and 1:20 (m/m). (c) From group one to group eight of agarose electrophoresis of DEP-siRNA when siRNA:DEP = 400:18.75, 400:37.5, 400:75, 400:150, 400:300, 400:600, 400:1200, and 400:2400 (m/m, ng).

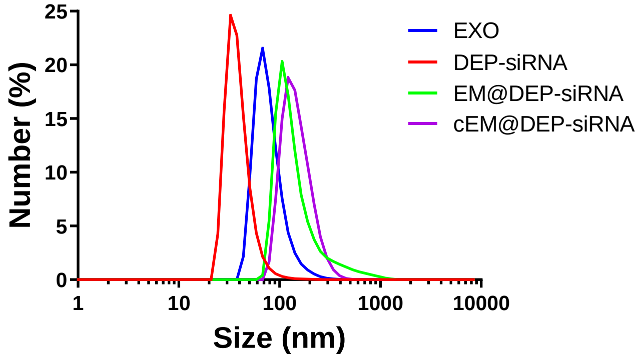

Figure S4. The particle size distribution of EXO, DEP-siRNA, EM@DEP-siRNA, and cEM@DEP-siRNA.

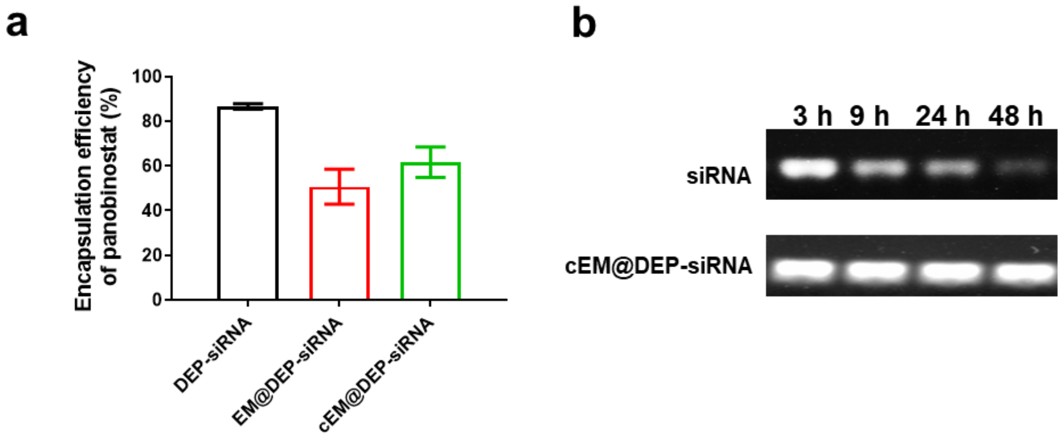

Figure S5. **Characteristics of exosomes drug delivery system.** (a) Encapsulation efficiency of panobinostat in DEP-siRNA, EM@DEP-siRNA, and cEM@DEP-siRNA. (b) Agarose electrophores of siRNA and cEM@DEP-siRNA after incubated with 10% fetal bovine serum in PBS for 3 h, 9 h, 24 h, and 48 h.

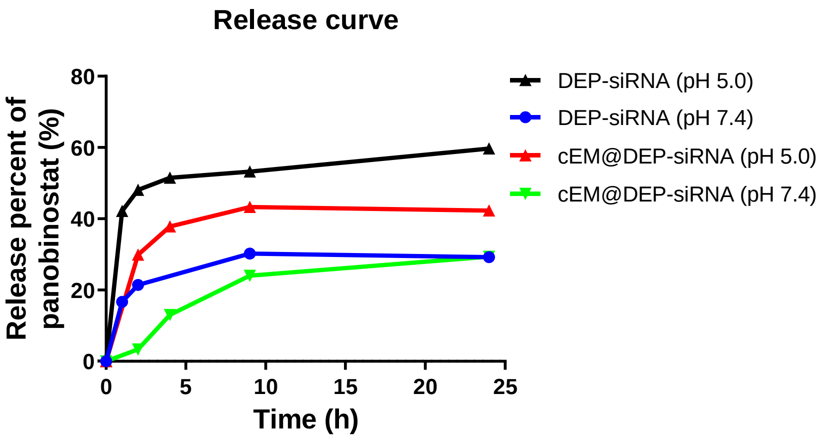

Figure S6. Drug release profiles of Pano in DEP-siRNA (pH 5.0, pH 7.4), and cEM@DEP-siRNA (pH 5.0, pH 7.4).

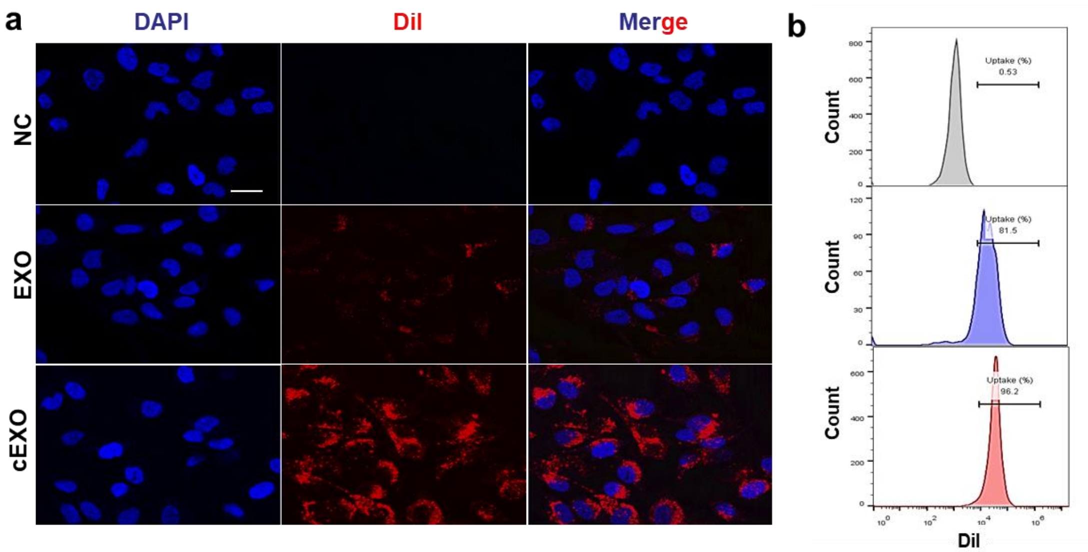

Figure S7. **The uptake of EXO and cEXO by DIPG cells**. (a) CLSM images of DIPG17 cells after co-incubation with Dil labeled EXO and cEXO for 4 h, scale bar: 20 μm. (b) The histogram of DIPG17 cells after co-incubation with Dil labeled EXO and cEXO for 4 h.

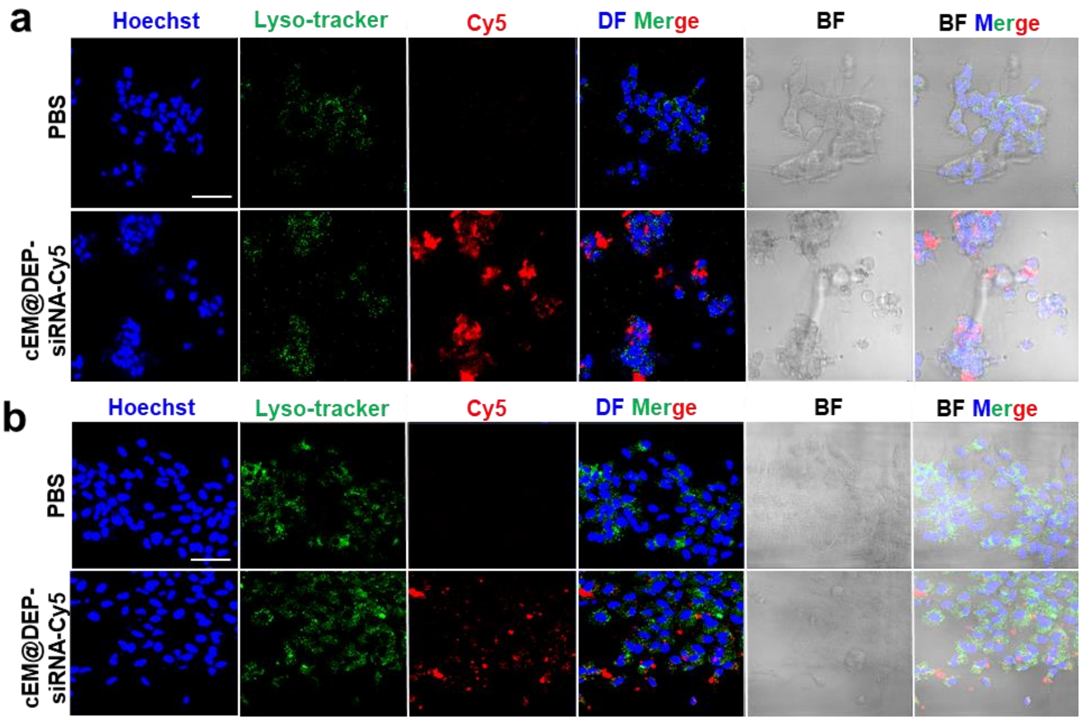

Figure S8. Endosomal escape of cEM@DEP-siRNA in DIPG cells observed by confocal

laser scanning microscopy (CLSM). TT150630 cells (a) and TT150714 cells (b) were incubated with cEM@DEP-siRNA-Cy5 for 4 h and the distribution of siRNA-Cy5 in DIPG cells was observed by CLSM. Blue, nucleus; green, endosomes stained with Lyso-Tracker Green; red, siRNA-Cy5. DF, dark field; BF, bright field. Scale bar: 50 μm.

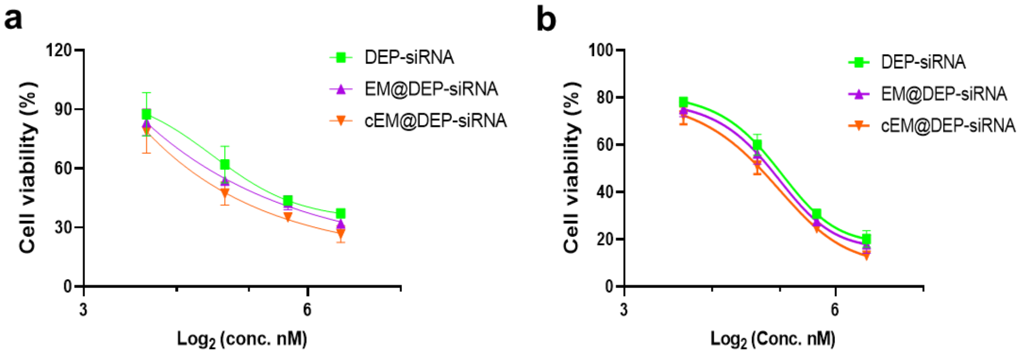

Figure S9. Cell viability of DIPG (a) TT150630 and (b) TT150714 after 72 h of different treatment groups by Cell Titer-glo.

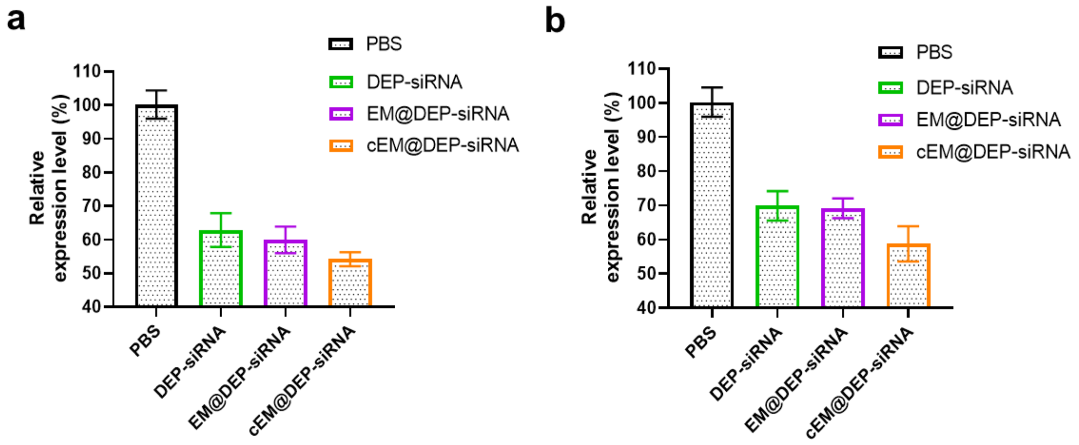

Figure S10. Relative expression levels of PPM1D mRNA in TT150630 (a) and TT150714 (b) cells after treated by PBS, DEP-siRNA, EM@DEP-siRNA, and cEM@DEP-siRNA for 72 h.

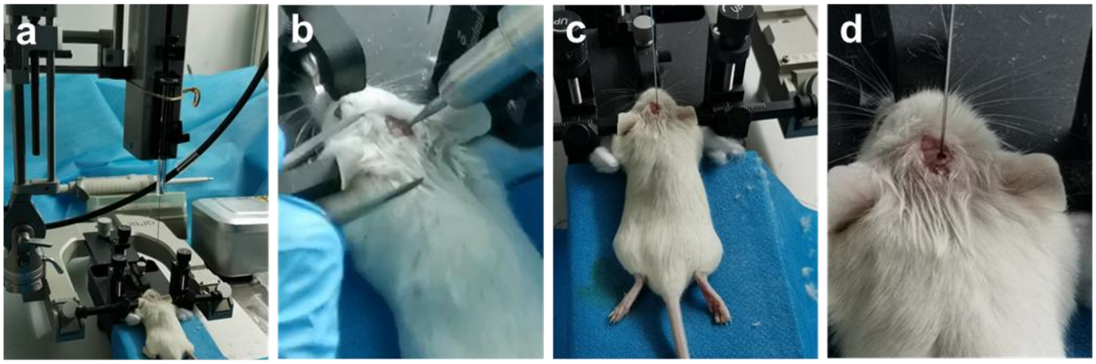

Figure S11. Operation process of constructing orthotopic DIPG model mice. (a) After successful anesthesia, NCG mice was fixed by a stereotactic fixator, and cut off the head hair, make a longitudinal incision by a scissor to expose the lambdoid suture; (b) Scrap the local periosteum of the lambdoid suture, and drill a hole (diameter of 1 mm) in the skull: the drill entry point chose 1.5 mm on the right side of the midline and 1.5 mm after the lambdoid suture; (c) About 100000 DIPG cells with Luciferase gene were suspended in 5 μL PBS and i slowly inject into the brainstem; (d) After the injection completed, stay locally for 2 minute and then slowly pull out the injection needle.

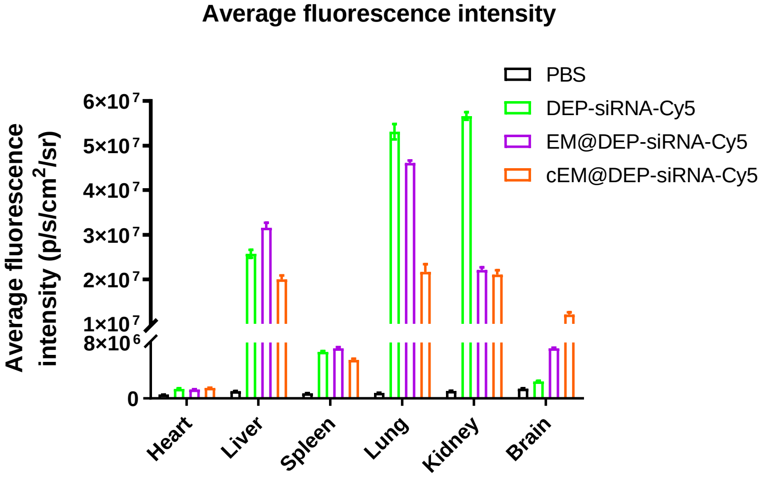

Figure S12. The average fluorescence of major organs (heart, liver, spleen, lung, kidney, and brain) at 12 h post-injection.

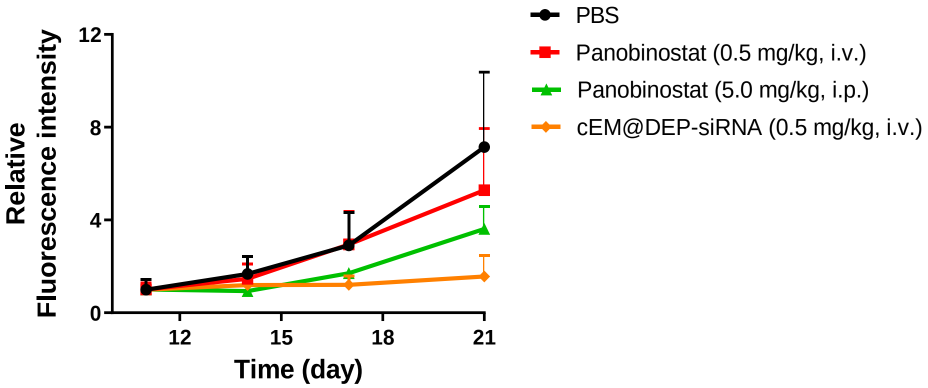

Figure S13. Bioluminescence signal intensity curves of different treatment groups at different time points in tumor site (n = 6).

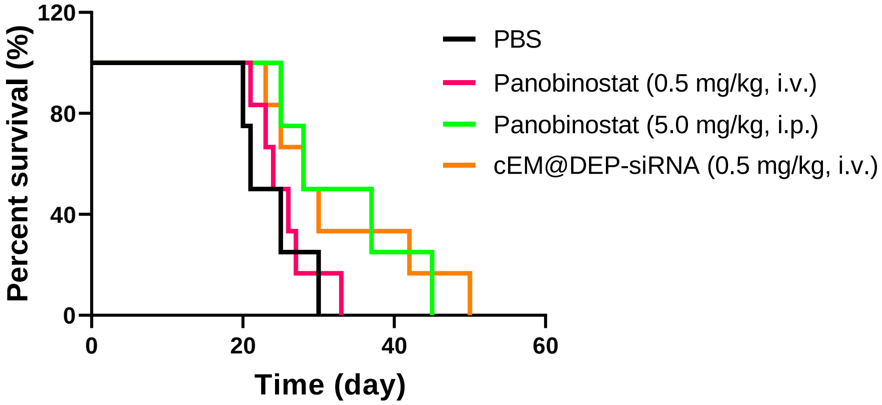

Figure S14. Kaplan-Meier survival curve of DIPG mice after treatment with different groups (n = 6).

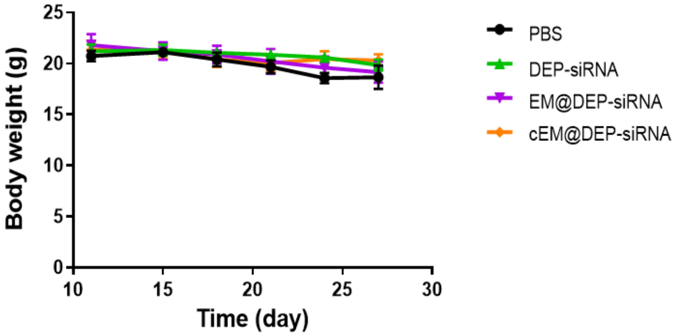

Figure S15. The body weight of DIPG mice after treatment with PBS, DEP-siRNA,

EM@DEP-siRNA, and cEM@DEP-siRNA (n = 6).

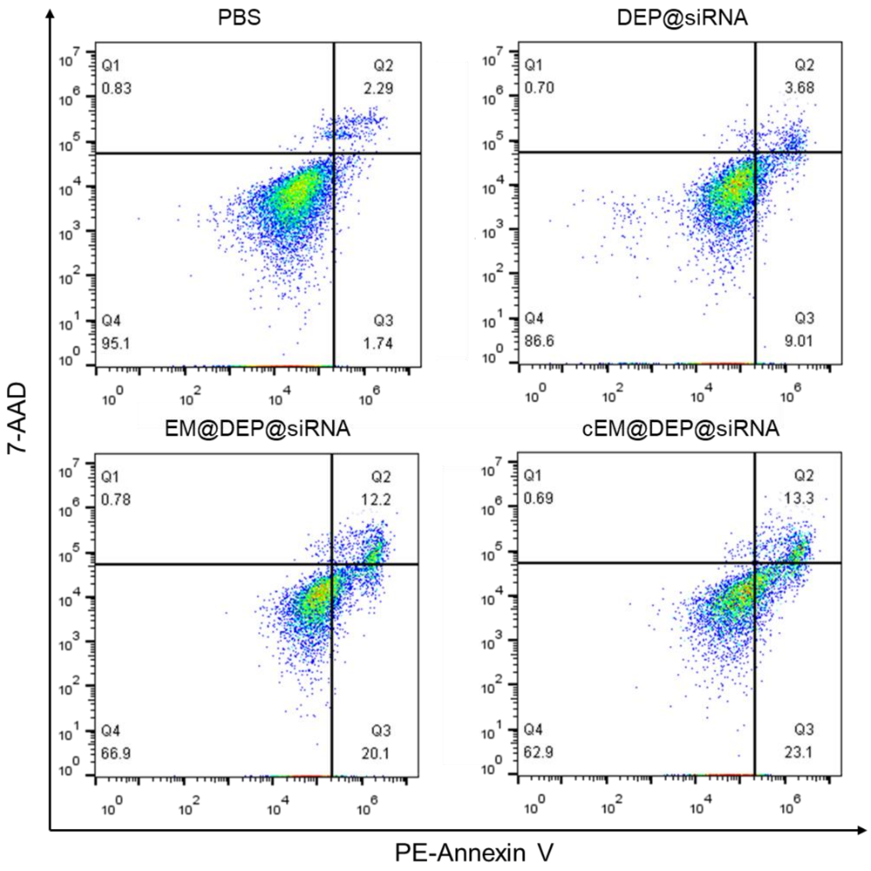

Figure S16. **The apoptosis of DIPG TT150714 cells measured by flow cytometry after 48 h treatment with different groups (n = 3).** The administered concentration of panobinostat and PPM1D-siRNA was 20 nM and 50nM, respectively, in all three formulations

Table S1. The sequences of PPM1D siRNA.

Name	Sequence（5’-3’）	
hs-PPM1D siRNA 1	Sense	GCAAGAGCACUUGGUGAUUdTdT
	Antisense	AAUCACCAAGUGCUCUUGCdTdT
hs-PPM1D siRNA 2	Sense	CCACAAGAUGCCAUCUCAAdTdT
	Antisense	UUGAGAUGGCAUCUUGUGGdTdT
hs-PPM1D siRNA 3	Sense	GGACAUUAGAAGAGUCCAAdTdT
	Antisense	UUGGACUCUUCUAAUGUCCdTdT
Scrambled siRNA	Sense	UUCUCCGAACGUGUCACGUdTdT
	Antisense	ACGUGACACGUUCGGAGAAdTdT
